# Phenotype-Specific Transcriptomic Responses to Glucocorticoid Signaling in the Prefrontal Cortex and Dorsal Raphe Nucleus Following Chronic Social Stress

**DOI:** 10.3390/ijms27146442

**Published:** 2026-07-20

**Authors:** Polina Ritter, Anastasiia Shuliupova, Vasiliy Reshetnikov, Natalia Bondar

**Affiliations:** 1 Institute of Cytology and Genetics (ICG), Siberian Branch of Russian Academy of Sciences (SB RAS), Prospekt Akad. Lavrentyeva 10, 630090 Novosibirsk, Russia; kisaretova@bionet.nsc.ru (P.R.);; 2 Translational Medicine Research Center, Sirius University of Science and Technology, Sirius Federal Territory, 354340 Sochi, Russia

**Keywords:** chronic stress, HPA axis, dexamethasone, glucocorticoid signaling, RNA-seq, prefrontal cortex, dorsal raphe nucleus, stress resilience

## Abstract

Chronic stress produces marked individual differences in behavioral adaptation and glucocorticoid sensitivity, but the molecular basis of this variability remains poorly understood. Here, we examined transcriptional responses to glucocorticoid receptor activation after chronic social defeat stress (CSDS) in male C57BL/6J mice. Based on behavioral responses during social interaction, stressed animals were classified into active coping strategy (AS) and passive coping strategy (PS) phenotypes. A total of 24 h after the final stress session, mice received 2 µg/g of dexamethasone (DEX) or a saline injection, and transcriptomic profiling of the prefrontal cortex (PFC) and dorsal raphe nucleus (DRN) was performed 6 h later using RNA sequencing. Chronic stress induced pronounced phenotype- and region-specific transcriptional alterations. The PFC showed extensive stress-associated gene expression changes, particularly in PS animals, whereas the DRN displayed comparatively fewer differentially expressed genes. Functional enrichment analysis nevertheless revealed substantial pathway-level remodeling in both regions. PS animals exhibited extensive pathway reorganization in the DRN, while AS animals showed broader suppression of enriched biological pathways in the PFC. Acute DEX administration further revealed marked phenotype-dependent differences in glucocorticoid-responsive transcriptional programs, with the strongest response observed in AS animals. Together, these findings demonstrate that chronic stress establishes distinct transcriptional states that shape subsequent DEX-induced transcriptional responses in a phenotype- and brain region-specific manner. Our results provide new insight into the molecular mechanisms underlying heterogeneity in stress adaptation.

## 1. Introduction

Stress is a fundamental adaptive response that enables organisms to cope with environmental challenges; however, chronic stress leads to profound and often maladaptive changes in both peripheral systems and the brain. A central mediator of these effects is the hypothalamic–pituitary–adrenal (HPA) axis, which regulates glucocorticoid secretion and coordinates systemic and neural responses to stress. Glucocorticoids act on virtually all brain regions, modulating transcriptional programs, synaptic plasticity, and cellular homeostasis. Under chronic stress conditions, however, this system becomes dysregulated, resulting in altered glucocorticoid feedback sensitivity and impaired glucocorticoid receptor (GR) signaling.

Accumulating evidence indicates that chronic stress does not induce a uniform biological state but instead gives rise to multiple adaptive trajectories characterized by distinct patterns of HPA-axis regulation and glucocorticoid sensitivity. Both clinical and preclinical studies indicate that individuals exposed to similar stressors may develop markedly different behavioral and physiological outcomes, ranging from vulnerability to resilience. This variability is paralleled by differences in HPA-axis function, including altered basal glucocorticoid levels and impaired negative feedback. While the dexamethasone suppression test (DST) has long been used to assess HPA-axis integrity, its inconsistent predictive value suggests that it captures only part of a more complex, multidimensional stress-response landscape.

In our previous work, we addressed this heterogeneity by characterizing HPA-axis responsiveness to synthetic glucocorticoid dexamethasone (DEX) in mice subjected to chronic social defeat stress. Using DEX as a probe of feedback sensitivity, we demonstrated that chronically stressed animals segregate into distinct response clusters defined by their basal corticosterone levels and degree of DEX-induced suppression [1]. Importantly, these clusters were not only endocrinologically distinct but also exhibited divergent behavioral phenotypes and differential expression of HPA axis-related genes across the hypothalamus and adrenal glands. These findings indicate that chronic stress gives rise to multiple, biologically meaningful adaptive trajectories rather than a single pathological state.

Consistent with this, we and others have shown that chronic stress disrupts GR-dependent regulation at multiple levels of the HPA axis, leading to altered gene expression responses to DEX and suggesting the development of glucocorticoid resistance [2,3,4,5,6]. Moreover, longitudinal analyses indicate that glucocorticoid-sensitive transcriptional programs are initially activated during early stress exposure but become attenuated with prolonged stress, pointing to a dynamic reorganization of GR signaling over time [7]. Together, these findings suggest that variability in DEX responsiveness reflects underlying differences in GR signaling capacity and feedback regulation.

At the neural level, chronic stress induces highly region- and phenotype-specific molecular changes. In established models such as chronic social defeat stress, susceptible and resilient individuals show distinct transcriptional and epigenetic signatures across multiple brain regions, including the prefrontal cortex (PFC) and dorsal raphe nuclei (DRN). Susceptibility is often associated with widespread transcriptional dysregulation, particularly in pathways related to synaptic function and neuronal plasticity, whereas resilience may involve more constrained or adaptive transcriptional responses [8,9]. Importantly, these patterns are not uniform: depending on the brain region and experimental context, resilient phenotypes may exhibit either enhanced or more tightly regulated gene expression programs, suggesting active and region-specific adaptation mechanisms.

The PFC and DRN are particularly relevant in this context. The PFC serves as a key hub for cognitive control and regulation of stress responses and is highly sensitive to chronic stress-induced alterations in excitatory/inhibitory balance and transcriptional regulation [10]. The DRN, as the primary source of serotonergic projections to the forebrain, plays a central role in shaping coping strategies and affective behavior, with serotonergic activity directly influencing stress-related behavioral outcomes [11]. Both regions are well-established targets of glucocorticoids and are tightly integrated with the HPA-axis function.

Despite these advances, a critical gap remains: it is still unclear how systemic differences in HPA-axis regulation translate into region-specific molecular responses to glucocorticoids in the brain. In particular, it is unknown whether animals with distinct HPA- axis response profiles also differ in their transcriptional sensitivity to glucocorticoids across brain regions.

Here, we address this question by focusing on DEX as a controlled and mechanistically interpretable probe of glucocorticoid signaling. Building on our previous demonstration of distinct HPA-axis response phenotypes, we hypothesize that these phenotypes will exhibit differential transcriptional responses to DEX in stress-relevant brain regions. Specifically, we propose that variability in systemic glucocorticoid sensitivity will be reflected in region-specific patterns of gene regulation in the PFC and DRN.

In this study, we analyze transcriptomic changes in the prefrontal cortex and dorsal raphe nucleus following chronic stress and 6 h after DEX administration. By integrating stress exposure with pharmacological activation of glucocorticoid signaling, we aim to identify molecular pathways that differentially respond to glucocorticoids across stress-response phenotypes. This approach allows us to directly probe the functional sensitivity of brain regions to glucocorticoid regulation, providing new insight into the mechanisms underlying heterogeneity in stress adaptation.

## 2. Results

### 2.1. Behavioral Stratification Defines Passive and Active Coping Phenotypes

**Figure 1:**
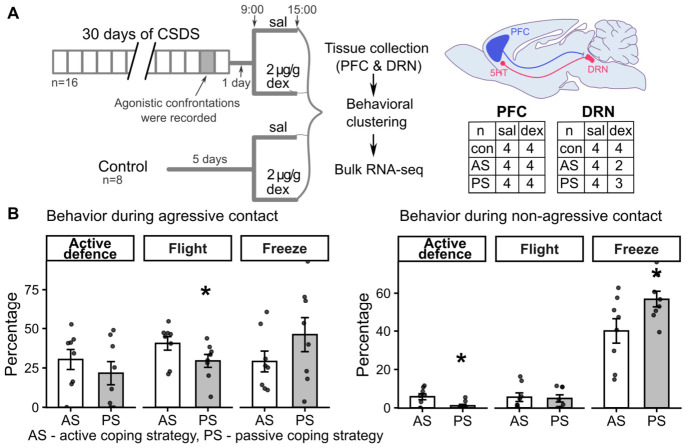
Experimental design and coping strategy stratification in chronic social defeat stress. (**A**) Experimental design. Male mice were randomly assigned to control or chronic social defeat stress (CSDS) groups. Stressed animals were subjected to daily agonistic confrontations for 30 days. Behavioral assessment during the agonistic confrontation test on day 29 was used for clustering of stressed animals into active coping strategy (AS) and passive coping strategy (PS) subgroups. A total of 24 h after the final stress exposure, animals received intraperitoneal injections of dexamethasone or saline, followed by tissue collection 6 h later. Prefrontal cortex and dorsal raphe nucleus samples were used for RNA-seq analysis. Tables on the right indicate the number of RNA-seq libraries generated for each experimental group and brain region. (**B**) Context-dependent behavioral profiles of PS and AS mice in the agonistic confrontation test. Behavioral parameters (% time) were analyzed during attacks and non-aggressive contact with the aggressor. Clustering based on behavior during non-aggressive contact identified two subgroups of stressed mice: PS (passive, increased freezing) and AS (active, increased escape and active defensive postures). Data are shown as mean ± SEM; statistical comparisons were performed using two-tailed Mann–Whitney U tests (*) (n = 8 per group). AS—active copying strategy, PS—passive copying strategy.

Using behavioral profiling in the agonistic confrontation test followed by clustering of responses during non-aggressive contact, we identified two subgroups of stressed mice that differed in coping strategy: PS (passive coping strategy) and AS (active coping strategy). AS animals exhibited significantly higher escape during partner attacks and increased active behaviors with reduced freezing during non-aggressive contact compared to PS (Mann–Whitney U test, *p* < 0.05), whereas PS mice showed the opposite pattern, characterized by predominant passive behavior (freezing) (Figure 1B).

### 2.2. Distinct Transcriptional Responses to Chronic Stress and Dexamethasone

#### 2.2.1. Chronic Stress-Induced Differential Expression

**Figure 2:**
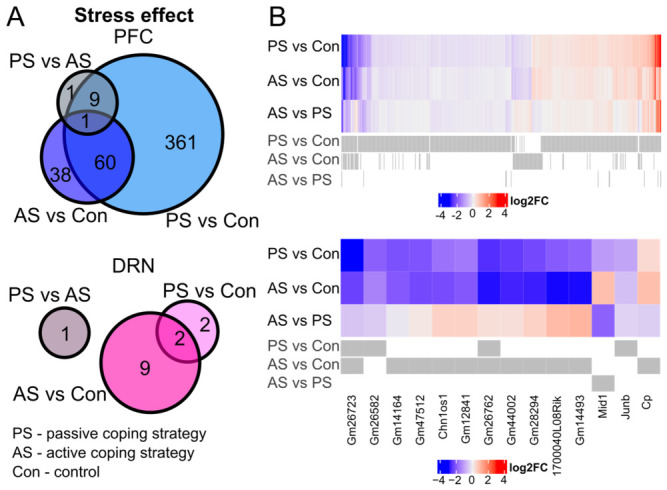
Differential gene expression induced by chronic stress across coping phenotypes. (**A**) Venn diagrams illustrate overlap of differentially expressed genes (DEGs; adjusted *p* < 0.1) in the PFC or DRN identified in saline-treated passive coping (PS vs. Con) and active coping (AS vs. Con) groups relative to saline-treated controls. (**B**) Heatmaps of the combined DEG lists for each region. Gray annotation bars below the heatmaps indicate in which specific comparison each gene reached statistical significance.

We assessed stress-induced transcriptional alterations in the PFC and DRN across the two subgroups of stressed mice, AS and PS, to determine how distinct behavioral responses relate to gene expression changes (Figure 2).

In the PFC, gene expression alterations induced by chronic stress were markedly more pronounced in PS animals, with 431 DEGs relative to control, compared to 99 DEGs in AS. Only 11 genes differed between PS and AS, indicating that chronic stress elicited largely concordant transcriptional changes in both groups, while the magnitude of these changes was substantially greater in PS.

In the DRN, chronic stress was associated with a substantially smaller number of DEGs compared to the PFC. In AS, 11 genes were differentially expressed relative to control, whereas only 4 DEGs were detected in PS. Direct comparison between PS and AS revealed differential expression of a single gene (*Mid1*).

#### 2.2.2. Glucocorticoid Responsiveness Across Coping Phenotypes

**Figure 3:**
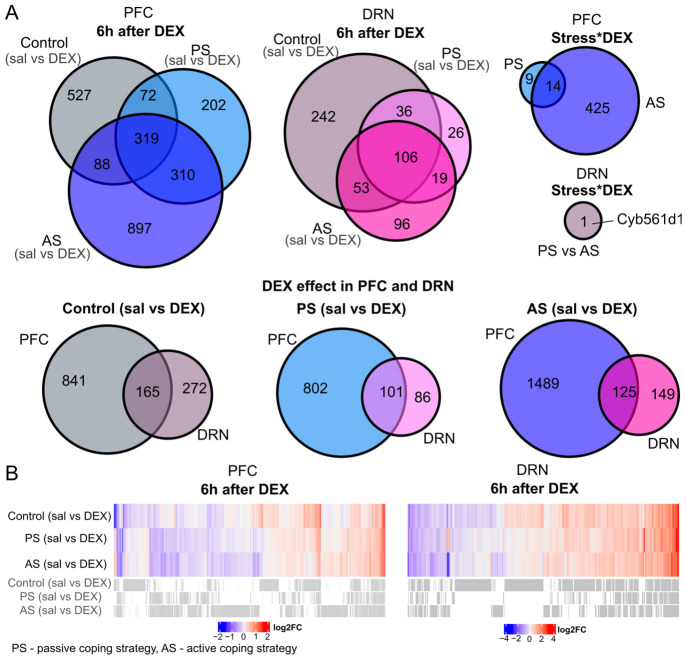
Differential gene expression induced by dexamethasone (6 h) across coping phenotypes. (**A**) Venn diagrams illustrate overlap of differentially expressed genes (DEGs; adjusted *p* < 0.1) in the PFC and DRN identified by comparing dexamethasone- and saline-treated animals within control (DEX vs. Sal), passive coping (PS DEX vs. Sal), and active coping (AS DEX vs. Sal) groups. (**B**) Heatmaps of the combined DEG lists for each region. Gray annotation bars below the heatmaps indicate in which specific comparison each gene reached statistical significance.

Next, we examined transcriptional responses to dexamethasone 6 h after administration in the PFC and DRN by comparing DEX- and saline-treated animals within control, PS, and AS groups (Figure 3).

Consistent with basal stress effects, DEX-induced transcriptional changes were substantially more pronounced in the PFC than in the DRN across all groups.

In control animals, DEX induced differential expression of 1006 genes in the PFC. In contrast to basal stress-related effects, the magnitude of the DEX response in the PFC was smaller in PS animals (903 DEGs) but larger in AS animals (1614 DEGs).

Analysis of the stress × DEX interaction term revealed that PS largely preserved a control-like transcriptional response, with only 23 interaction DEGs. In contrast, AS exhibited a markedly altered interaction profile, with 439 genes showing a DEX response significantly different from control, indicating a dysregulated glucocorticoid-dependent transcriptional program.

In the DRN, DEX elicited a weaker transcriptional response compared to the PFC. Control animals exhibited 437 DEX-responsive genes, whereas the number of DEGs was smaller in both PS (187 genes) and AS (274 genes).

Notably, stress × DEX interaction analysis identified only a single differentially expressed gene in the DRN (*Cyb561d1*), suggesting that glucocorticoid-dependent transcriptional regulation in this region is relatively preserved across stress-defined phenotypes.

Within each group and across brain regions, overlapping DEX-responsive genes predominantly exhibited unidirectional expression changes. Exceptions to this pattern were limited: in control animals (*Hrh1*, *Slit2*, *Adra2a*), in PS (*Hrh1*, *Itgb8*, *2900052N01Rik*), and in AS (*Hrh1*, *Ugt8a*, *Crocc*). Although *Hrh1* (Histamine Receptor H1) was identified as a DEX-responsive DEG across all groups, its regulation was region-specific: DEX consistently downregulated *Hrh1* expression in the PFC but upregulated it in the DRN. This opposing pattern suggests distinct functional roles of histaminergic signaling in these regions. Increased *Hrh1* expression in the DRN may enhance neuronal excitability, whereas reduced expression in the PFC may attenuate excitatory drive, potentially dampening stress-related responses.

#### 2.2.3. Phenotype-Specific Gene Ontology Enrichment of Unique DEGs

Given that most shared DEGs changed in the same direction across groups, we performed GO enrichment analysis on group-specific genes to identify stress- and DEX-associated molecular signatures unique to each phenotype.

#### 2.2.4. Chronic Stress Effect

**Figure 4:**
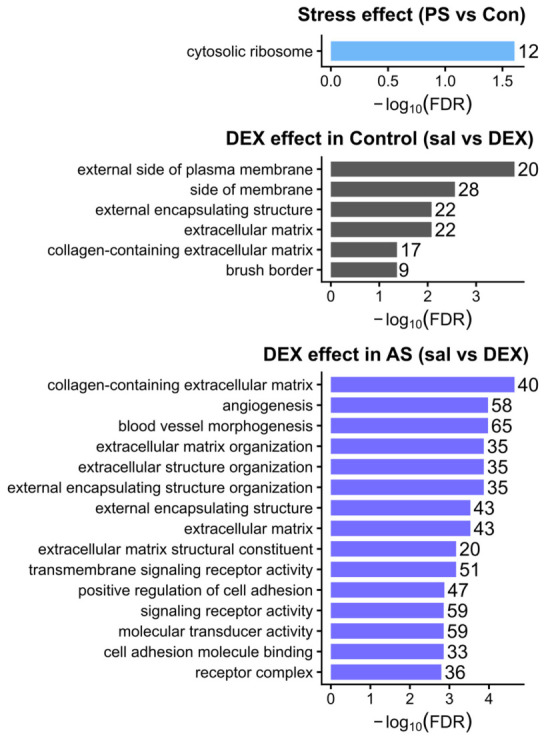
Gene Ontology enrichment of phenotype-specific differentially expressed genes in PFC. GO analysis was performed on unique DEGs associated with stress effects (PS vs. Con and AS vs. Con under saline conditions) and dexamethasone effects (DEX vs. Sal within PS and AS groups) to identify phenotype-specific molecular signatures. Numbers adjacent to bars indicate the number of DEGs within each category. Bars are color-coded by comparison group. AS, active coping strategy; PS, passive coping strategy.

In the PFC, PS-specific genes (361 DEGs) were significantly enriched for cytosolic ribosome components, indicating altered translational regulation in response to stress (Figure 4). No significant GO or KEGG enrichment was detected for AS-specific genes (38 DEGs).

In the DRN, due to the low number of DEGs (two in PS and nine in AS), GO enrichment analysis did not reveal robust pathway-level effects, as most categories were driven by single-gene annotations. Instead, transcriptional changes were primarily represented by the immediate-early gene *Junb* in PS and metalloprotein ceruloplasmin gene *Cp* in AS.

#### 2.2.5. Dexamethasone Effect in the PFC

In control animals (527 unique DEGs), DEX induced the enrichment of genes associated with extracellular and membrane-related compartments, including the external side of the plasma membrane and collagen-containing extracellular matrix. KEGG analysis additionally identified sphingolipid metabolism (Figure 4).

In PS animals (202 unique DEGs), no significant GO or KEGG enrichment was detected. Consistent with this, most PS DEGs overlapped with controls and changed in the same direction, and the stress × dexamethasone interaction yielded minimal additional transcriptional effects.

In contrast, PFC of AS animals (897 unique genes) exhibited robust enrichment for genes related to extracellular matrix organization, angiogenesis, and blood vessel morphogenesis. KEGG pathways included cell adhesion and signaling-related processes (e.g., IgSF CAM signaling, calcium signaling, adherens junctions, and Notch signaling), indicating enhanced extracellular remodeling and intercellular communication.

#### 2.2.6. Dexamethasone Effect in the DRN

In the DRN, DEX did not produce significant GO enrichment in any group. KEGG associations were nominal and did not reach statistical significance. Transcriptional changes were limited and gene-specific, including growth factor-related genes (e.g., *Igf1r*, *Pdgfra*) and neurotransmitter-associated genes (e.g., *Grin2c*, *Htr5b*), without coherent pathway-level organization.

Overall, GO analysis of group-specific DEGs revealed relatively modest and phenotype-dependent pathway enrichment, particularly in the DRN, suggesting that broader transcriptional reorganization may be better captured by gene set level approaches.

### 2.3. Gene Set Enrichment Analysis Reveals Coping Strategy-Dependent Remodeling of Cortico-Raphe Circuits

To further characterize the functional relevance of the observed transcriptional changes, we performed Gene Set Enrichment Analysis (GSEA) on the ranked gene expression profiles. While GO enrichment of uniquely responsive DEGs provided subgroup-specific insights, it was inherently limited by the size of discrete gene lists. Because stress-related transcriptomic responses are often distributed across many genes with small effect sizes rather than driven by large changes in individual genes, GSEA enables a more sensitive detection of coordinated, pathway-level alterations across the entire transcriptome, thereby providing a more comprehensive characterization of the underlying biological processes.

#### 2.3.1. Chronic Stress Effect

**Figure 5:**
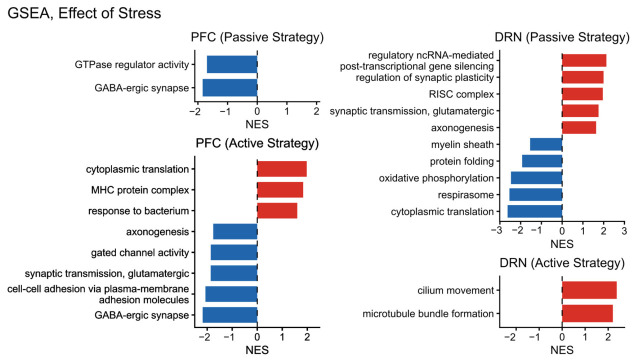
Gene Set Enrichment Analysis (GSEA) reveals stress-associated alterations in functional gene programs. Panels show selected enriched pathways in the PFC and DRN under stress conditions (FDR < 0.05). Bars represent Normalized Enrichment Scores (NES) for selected categories. Positive NES (red) indicate upregulated pathways, negative NES (blue) indicate downregulated pathways.

In the PFC, chronic stress induced limited changes detected by GSEA in PS animals, with only two significantly downregulated gene sets identified (Figure 5), including GABAergic synapse and regulation of GTPase activity pathways.

In contrast, AS animals exhibited a broader stress-associated transcriptional remodeling in the PFC, with 30 significantly enriched gene sets, largely downregulated and partially overlapping with PS (e.g., GABAergic synapse). However, AS uniquely showed suppression of additional synaptic and structural pathways, including axonogenesis, glutamatergic synaptic transmission, cell adhesion and gated channel activity, suggesting more extensive cortical synaptic remodeling.

Six gene sets were upregulated in PFC of AS animals, primarily related to immune and translational processes (e.g., response to bacterium, MHC protein complex, cytoplasmic translation).

In PS animals, chronic stress induced extensive transcriptional reorganization in the DRN, reflected by enrichment changes across 52 gene sets. Downregulated pathways (26) were primarily related to ribosomal and translational processes, oxidative metabolism, and myelination, indicating reduced biosynthetic and metabolic capacity. Conversely, upregulated gene sets in DRN of PS animals included axonogenesis, glutamatergic transmission, and synaptic plasticity, suggesting activation of plasticity-related signaling. Several of these pathways were suppressed in PFC of PS animals, highlighting opposite cortical-raphe regulation. Interestingly, in addition, among upregulated categories there are ncRNA-mediated gene silencing and RISC complex.

In contrast, AS animals displayed a restricted DRN response (nine gene sets), all upregulated and mainly associated with microtubule and cilia-related processes.

The cilium/axoneme-related enrichment observed in the DRN corresponded to a canonical motile cilia gene program, indicative of multiciliated ependymal cells lining the cerebral aqueduct. This included key components of axonemal structure and motility, such as dynein motors (*Dnah5*, *Dnah9*), dynein regulatory complex proteins (*Drc1*, *Drc3*), radial spoke proteins (*Rsph1*, *Rsph4a*), and structural elements including tektins (*Tekt1*, *Tekt2*) and CFAP family proteins (*Cfap43*, *Cfap44*), which are not characteristic of neurons, which typically possess only primary (non-motile) cilia. In the brain, such coordinated gene expression is a hallmark of ependymal cells lining the ventricular system and aqueduct [12,13,14]. As all samples were collected using an identical microdissection strategy, the stress-dependent enrichment of this signature likely reflects condition-specific differences in the contribution or transcriptional activity of periaqueductal ependymal cells, rather than intrinsic neuronal changes.

Overall, chronic stress induced coping strategy-dependent and region-specific molecular adaptations: PS animals showed limited cortical but extensive DRN reorganization, whereas AS animals exhibited broader cortical suppression and a restricted, structurally oriented DRN response.

#### 2.3.2. Effects of Dexamethasone

##### Dexamethasone Effect in the PFC

**Figure 6:**
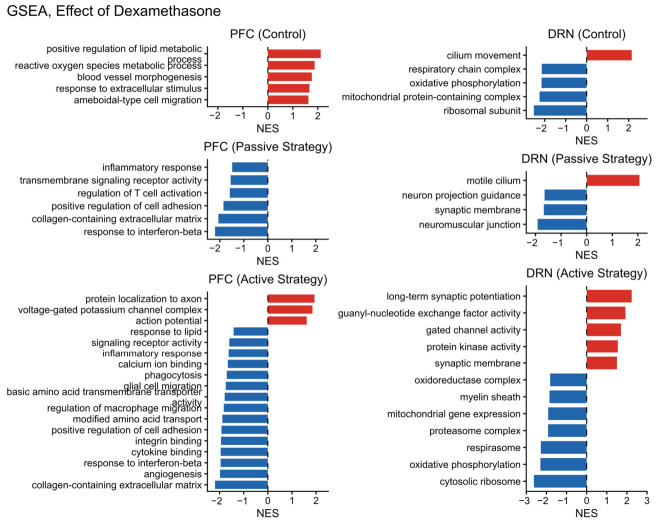
Gene Set Enrichment Analysis (GSEA) reveals dexamethasone-induced remodeling of functional gene programs. Panels show selected enriched pathways in the PFC and DRN following Dexamethasone treatment (FDR < 0.05). Bars represent Normalized Enrichment Scores (NES) for selected categories. Positive NES (red) corresponds to upregulated pathways, negative NES (blue) to downregulated pathways.

In control animals, DEX induced modest transcriptional changes at the level of functional gene programs in the PFC (eight significantly enriched gene sets, Figure 6), as assessed by GSEA of ranked gene-expression lists. Ribosomal pathways were downregulated, whereas upregulated categories included lipid-related processes, radical metabolism, angiogenesis, response to extracellular stimulus, and cell migration.

In PS animals, GSEA revealed that DEX triggered broad suppression in the PFC, with 32 significantly enriched gene sets identified, all of which were downregulated. These gene programs are predominantly involved in interferon signaling, antigen presentation, innate and adaptive immune responses, extracellular matrix organization, cell adhesion, and transmembrane signaling receptor activity. Many of these categories overlapped with AS animals and were regulated in the same direction.

AS animals showed the most extensive DEX response (89 categories), and the vast majority of programs were downregulated. Similar to PS, pathways suppressed by DEX included extracellular matrix, cell adhesion, angiogenesis, interferon and cytokine signaling, and adaptive immunity, but additionally extended to other immune-related categories, indicating a profound repression of immune and inflammatory programs. Other downregulated categories include amino acid transport, macrophage migration, glial migration, calcium ion binding, and phagocytosis.

Only four categories in DRN of AS animals were upregulated in response to DEX, including action potential, potassium channel activity, and protein transport to axon, suggesting a selective enhancement of neuronal excitability-related processes.

Overall, DEX predominantly suppressed immune and extracellular signaling pathways in stressed animals, with the strongest and most widespread effect observed in AS.

##### Dexamethasone Effect in the DRN

In controls, GSEA showed that DEX affected 14 gene sets in the DRN (Figure 6), with 10 of them downregulated and primarily related to ribosomes, translation, mitochondrial function, respiration, and oxidative phosphorylation. Upregulated categories included cilium/axoneme-related processes.

In PS animals, DEX induced changes in 12 gene sets in DRN, as revealed by GSEA. Downregulated pathways included neuromuscular junction, synapse, and neuron projection. In contrast, upregulated categories were mainly associated with cilium and microtubules. Notably, synapse-related processes were commonly affected in both PS and AS, but in opposite directions (down in PS, up in AS).

AS animals exhibited a broader DEX response in DRN (25 gene sets). Seventeen were downregulated, including ribosomal and translational processes, mitochondrial respiration, proteasome, myelin sheath, and oxidoreductase complex, indicating suppression of energy metabolism and protein turnover. Upregulated categories included synapse, protein kinase activity, ion channel activity, long-term synaptic transmission, and guanine nucleotide exchange factor activity, consistent with activation of signaling and synaptic modulation pathways.

In summary, acute glucocorticoid exposure produced phenotype- and region-specific effects. In the PFC, DEX predominantly suppressed immune-related pathways, especially in AS animals. In the DRN, DEX reduced metabolic and translational programs in controls and AS, while differentially modulating synaptic processes in PS and AS, reinforcing the idea of coping strategy-dependent molecular responsiveness to glucocorticoids.

### 2.4. Cell-Type Specific Genes Enrichment Analysis

Bulk RNA-seq does not allow direct identification of the cellular origin of transcriptional changes. However, to gain insight into which cell types might contribute to the observed differential expression, we tested whether DEGs were enriched for known cell-type-specific marker genes [15]. The analysis was performed using DEG lists obtained from stress-related comparisons (PS vs control and AS vs control) and dexamethasone-related comparisons (DEX vs saline within each group). Enrichment was evaluated using odds ratio (OR) and Fisher’s exact test, with the background defined as all genes tested for differential expression.

Chronic stress alone did not elicit cell-type specific transcriptional changes in the PFC. However, DEX-induced DEGs were significantly enriched for markers of specific cell populations, suggesting a cell-type-specific response to glucocorticoid stimulation.

**Figure 7:**
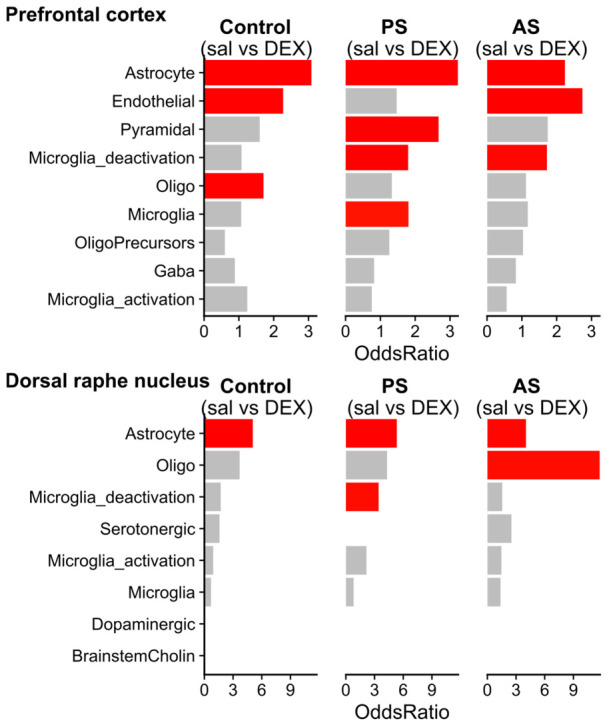
Enrichment of DEG sets (*p* adj < 0.1) with cell-type-specific genes. The *x*-axis shows the odds ratio (OR) as a measure of enrichment. Significant enrichments (*p* < 0.05) are marked in red; non-significant changes (*p* > 0.05) are shown in gray. AS, active coping strategy; PS, passive coping strategy.

In control animals, DEX triggered pronounced cell-type specific effects in the PFC, including enrichment of astrocyte markers (68.7% of DEGs upregulated), endothelial markers (96.8% up), and oligodendrocyte markers (90% down) (Figure 7).

In PS animals, DEX-induced DEGs were enriched in pyramidal neurons (six genes, 83% up), deactivated microglia (24 genes, 83% down), microglia (38 genes, 66% down), and astrocytes (63 genes, 64% down). The predominance of downregulated microglial and astrocytic markers is consistent with the strong suppression of interferon, antigen presentation, and inflammatory pathways identified by GSEA, indicating glucocorticoid-mediated immune silencing in this phenotype.

In AS animals, the strongest enrichment was observed for deactivated microglia (41 genes, 88% down), endothelial cells (60 genes, 97% down), and astrocytes (78 genes, 69% down). This closely mirrors the extensive downregulation of extracellular matrix, cytokine, adhesion, and angiogenesis pathways detected in GSEA, suggesting coordinated suppression of neurovascular and immune-associated programs in AS.

In contrast to the PFC, cell-type enrichment in the DRN was limited across conditions and without convergence on strong vascular signatures as observed in the PFC. Chronic stress alone led to a small number of DEGs in the DRN, insufficient to detect cell-type enrichment.

In control animals, DEX-responsive genes were enriched only for astrocytes, with a roughly equal proportion of up- and downregulated genes. In PS, DEX-responsive DEGs were enriched in deactivated microglia (six genes, 50% up, 50% down) and astrocytes (nine genes, 89% up). In AS, enrichment was observed in oligodendrocytes (four genes, 100% down) and astrocytes (10 genes, 70% up). Notably, no enrichment was observed for serotonergic neurons; the only DEG in this category was *Htr5b*, which was downregulated in response to DEX in AS.

## 3. Discussion

The present study reveals pronounced phenotype- and region-specific transcriptional responses to chronic stress and acute GR activation. Chronic stress induced extensive transcriptional changes in the PFC, particularly in PS animals, showing passive coping phenotype, whereas the DRN exhibited comparatively modest gene expression alterations. At the pathway level, however, stress reorganized functional programs differently across phenotypes: PS animals showed extensive pathway remodeling in the DRN, while AS animals, which exhibit active coping phenotype, displayed broader pathway suppression in the PFC. Acute DEX exposure subsequently revealed strong phenotype-dependent differences in GR-dependent transcriptional responsiveness, with the most extensive response observed in AS animals. Together, these results suggest that chronic stress establishes distinct baseline transcriptional states that shape the magnitude and organization of downstream GR-dependent transcriptional programs.

### 3.1. Phenotype-Dependent Pathway Re-Organization After Chronic Stress

Coping strategies in rodents are commonly defined as active or passive behavioral responses across stress paradigms. In the forced swim and tail suspension tests, active coping is reflected by escape-directed behavior and mobility, whereas passive coping is characterized by immobility [16,17]. In social defeat models, a similar distinction is observed during interactions with an aggressor, where active coping involves escape and reduced freezing, while passive coping is associated with freezing and behavioral withdrawal, often increasing with repeated stress exposure [7,18,19]. This behavioral stratification provides a relevant framework for interpreting the pronounced phenotype-specific differences observed at the transcriptomic level.

In the PFC, PS mice exhibit limited pathway remodeling despite a high number of DEGs, with downregulation of GABAergic synapse and GTPase activity pathways, consistent with reduced inhibitory control and behavioral inflexibility under chronic stress [7,20,21,22,23,24].

In contrast, AS animals exhibited broader pathway-level changes in the PFC, with suppression of multiple synaptic and structural processes including axonogenesis, glutamatergic transmission, and cell adhesion. This is consistent with previous transcriptomic studies showing that chronic stress produces broad gene expression alterations in the PFC, affecting synaptic signaling, metabolic processes, and translational pathways [7,9,21,23,25]. Interestingly, AS animals also displayed upregulation of immune-related and translational pathways, including response to bacterium, MHC protein complex, and cytoplasmic translation. Activation of immune signaling and translational processes in the PFC has been reported in stress transcriptomic studies and is often interpreted as reflecting neuroimmune activation, particularly involving astrocytes and microglia [26,27]. Additionally, upregulation of translational machinery has been observed in stress-exposed and stress-resilient mice, suggesting increased protein synthesis demands associated with cellular adaptation [23,24].

Although direct transcriptomic comparisons of active and passive coping phenotypes remain limited, analogous patterns have been described in studies of stress susceptibility and resilience. In CSDS models, animals segregate into stress-susceptible and stress-resilient subpopulations based on performance in the social interaction test (SIT), where susceptible animals exhibit reduced interaction time with an unfamiliar conspecific (social avoidance), while resilient mice maintain interaction levels comparable to controls [28,29]. Importantly, coping strategy and stress susceptibility represent related but distinct conceptual frameworks. The active/passive coping classification used here reflects individual differences in behavioral responses during social defeat, whereas resilience and susceptibility are typically defined by the degree of functional impairment or preservation of adaptive behaviors following chronic stress exposure. Therefore, although coping phenotypes may overlap with stress-resilient or stress-susceptible profiles described in other paradigms, the present classification should be interpreted as identifying coping-related phenotypic heterogeneity within the specific CSDS paradigm used in this study rather than as a direct equivalent of resilience or vulnerability.

In PFC, susceptibility is typically associated with a larger number of DEGs and dysregulation of synaptic and signaling pathways, whereas resilience involves more selective transcriptional changes and activation of specific adaptive processes [9,24,29,30]. For example, in SSTCre:γ2f/f mice, a genetic model of stress resilience, in which the γ2 subunit of the GABAA receptor is selectively deleted in somatostatin-positive (SST) interneurons, leading to reduced inhibitory input onto these cells (i.e., functional disinhibition of SST interneurons). Transcriptomic analysis of the mPFC, after chronic variable stress for 21 days in this model, revealed fewer stress-induced DEGs compared to controls, together with upregulation of genes involved in mRNA translation and ribosomal function, indicating increased protein synthesis as a core adaptive mechanism [24], which we also observed in AS animals. In contrast to resilient animals and in concordance with our observations of PS group, stress-susceptible animals in standard CSDS paradigms show broader transcriptional changes, including genes related to synaptic transmission, neuronal structure, and intracellular signaling [9,23]. Additional support for this framework comes from a recent study combining behavioral stratification with cell-type-specific translatome profiling in mPFC projection neurons [29]. Using a paradigm of repeated unpredictable stress across different genetic backgrounds (C57BL/6, BALB/c, DBA/2), the authors demonstrated that susceptibility is strongly strain-dependent and defined by both social avoidance (SIT) and anhedonia (SPT). Importantly, the most vulnerable behavioral subtype (combined social avoidance and anhedonia) exhibited the largest number of DEGs (1089) in anterior Paraventricular Nucleus of the Thalamus (aPVT)-projecting mPFC neurons, whereas resilient animals showed markedly fewer changes (85 DEGs). Mechanistically, stress vulnerability was linked to increased expression of the histone demethylase KDM5C in glutamatergic neurons, leading to demethylation of H3K4me3 and altered transcriptional regulation.

At the same time, not all studies report a simple increase in DEGs in susceptible animals. For example, after 5 days of unpredictable stress, in a nucleus-specific RNA-seq study using Arc-GFP mice to isolate recently activated neurons, susceptible mice showed increased expression of immediate early genes (e.g., *Fos*, *Arc*) and synaptic activity markers in the PFC, but only a small number of DEGs overall [31].

Although the present study focused on the PFC and DRN, the observed differences in glucocorticoid-responsive transcription are likely to represent only part of a broader network of stress-related adaptations. In addition to the PFC and DRN, other interconnected regions, including the hippocampus and amygdala, play essential roles in stress adaptation and affective regulation through reciprocal interactions with the HPA axis. The hippocampus contributes to inhibitory feedback regulation of the HPA axis and is highly susceptible to chronic stress-induced transcriptional and structural remodeling, whereas the amygdala promotes emotional processing, becomes hyperactive under chronic stress, and influences stress responses through reciprocal interactions with the PFC, DRN, and hypothalamic CRH circuitry [32,33].

In another study, after 10 days of CSDS and 48 h after last confrontation, resilient animals exhibited a stronger transcriptional response in PFC, as well as in amygdala, ventral hippocampus, and nucleus accumbens [30]. This pattern is consistent with our observations in AS mice, which exhibit the highest number of DEGs in the PFC after GR activation with DEX, reflecting enhanced inducible transcriptional plasticity.

Across these models, ribosomal and translational pathways emerge as a key point of divergence: resilience is repeatedly associated with upregulation of translation-related genes and increased protein synthesis capacity, whereas susceptibility is linked to either dysregulated or reduced translational activity [23,24].

Subcortical regions generally exhibit fewer DEGs compared to cortical areas following chronic stress, although they often show pronounced changes at the level of specific functional pathways and neuronal activity [28,30].

In the DRN, PS mice display extensive pathway-level reorganization, including downregulation of cytosolic ribosome, oxidative metabolism, and myelination-related processes, suggesting reduced biosynthetic and metabolic activity, and upregulation of axonogenesis and glutamatergic signaling pathways, indicating active structural remodeling. In contrast, AS mice show limited DRN pathway changes, primarily involving cytoskeletal dynamics rather than synaptic remodeling.

These findings are consistent with recent transcriptomic and epigenetic analyses of the DRN in CSDS model, which demonstrated that stress-susceptible mice exhibit widespread gene expression changes enriched for neuronal development, axon guidance, and synaptic transmission pathways, whereas resilient animals show minimal transcriptional alterations and remain closer to control profiles [8]. Importantly, susceptibility in this study was also associated with reduced levels of the H3K4me3Q5ser histone serotonylation mark, indicating impaired gene regulation in DRN.

Related patterns have been described in other subcortical regions. In mesolimbic circuits, including the VTA and NAc, stress-susceptible mice exhibit persistent increases in neuronal activity (e.g., elevated firing rates of VTA dopamine neurons) and upregulation of plasticity-related genes such as *Bdnf*, whereas resilient animals engage compensatory mechanisms that normalize excitability [28].

In this context, the PS phenotype in our study resembles stress susceptibility, as it is characterized by a higher number of baseline DEGs and enrichment of synaptic pathways. In contrast, AS animals show fewer baseline transcriptional alterations but a stronger GR-dependent response and upregulation of translational pathways in the PFC, consistent with a resilience-like profile involving preserved inducible plasticity and active protein synthesis.

### 3.2. GR-Dependent Transcriptional Responsiveness in PFC

Chronic stress and depressive-like states are known to induce dysregulation of the HPA-axis, leading to altered glucocorticoid sensitivity both in humans and in animal models. In rodents, chronic social defeat stress and other prolonged stress paradigms can produce phenotypic divergence in glucocorticoid responsiveness, with some animals developing resistance to glucocorticoids at the level of both HPA-axis feedback and neuronal transcriptional regulation [7,22,34].

In our previous studies, we showed that chronic social stress in C57BL/6 mice induces changes in HPA-axis function, including altered sensitivity to dexamethasone and dysregulation of GR-related gene expression [5,6]. Importantly, we also showed that these stress-induced alterations are not uniform: stressed animals segregate into distinct phenotypic groups characterized by differential basal corticosterone levels, dexamethasone responsiveness, and patterns of HPA-axis gene expression [1]. Within this framework, variability in GR-dependent transcriptional regulation may represent an important mechanism underlying individual differences in stress vulnerability and the development of depression-like states.

The 6 h timepoint likely captures a secondary, integrative phase of the glucocorticoid response. At this stage, immediate early GRE-driven targets have largely peaked, and transcriptional profiles are shaped by downstream transcription factor cascades, feedback regulators, and coordinated functional programs [5,35,36,37]. Notably, our previous data indicate that chronic stress alters not only the magnitude but also the temporal dynamics of glucocorticoid signaling, leading to a delayed and dysregulated transcriptional response to dexamethasone in both central and peripheral components of the HPA axis [5]. Within this framework, the transcriptional differences observed between PS and AS animals likely reflect the activation of distinct downstream regulatory programs following GR stimulation, such as metabolic, immune-modulatory, and plasticity-related pathways.

Acute DEX exposure revealed phenotype-dependent differences in GR-dependent transcriptional responsiveness. In AS animals, GR stimulation suppressed immune- and extracellular-signaling pathways in the PFC, consistent with activation of canonical glucocorticoid anti-inflammatory programs [35,36]. In PS mice, GR-dependent responses are markedly weaker, likely due to pre-engagement of transcriptional programs by chronic stress, consistent with the role of baseline chromatin state in shaping GR-binding and downstream transcriptional cascades [2,38,39].

A key aspect of the phenotype-specific DEX response involves differential engagement of glial and neurovascular transcriptional programs, as suggested by the cell-type enrichment observed in the results. In AS animals, GR activation predominantly affected astrocytic, microglial, and endothelial gene sets, consistent with the downregulation of immune, extracellular matrix, adhesion, and angiogenesis pathways. This indicates coordinated suppression of neuroimmune and neurovascular functions, potentially implicating modulation of blood–brain barrier integrity and glial support in the PFC [20,26,40].

In PS mice, DEX-responsive genes showed engagement of glial and endothelial programs and greater representation of neuronal categories, reflecting limited additional GR-dependent modulation on a background of stress-primed transcriptional activity. These observations are consistent with prior studies showing that GR signaling in the CNS preferentially targets glial and endothelial pathways, influencing cytokine signaling, matrix remodeling, and vascular regulation [41,42].

Overall, the stronger DEX-induced transcriptional responses in AS animals may result from lower baseline engagement of PFC transcriptional programs, whereas PS animals, with extensive stress-induced DEGs and upregulation of translation pathways at baseline, exhibit a constrained capacity for additional GR-dependent changes.

### 3.3. GR-Dependent Transcriptional Responsiveness in the DRN

In contrast to the PFC, DEX-induced transcriptional responses in the DRN were more modest and differed in their functional organization. Rather than strong regulation of immune and extracellular signaling pathways, GR activation in this region primarily affected programs related to cellular metabolism and synaptic signaling.

Phenotype-specific differences were most evident in synaptic pathways. In PS animals, DEX reduced expression of genes associated with synaptic organization and neuronal projections, whereas in AS animals synaptic categories, including ion channel activity and long-term synaptic signaling, were upregulated, suggesting increased transcriptional support for synaptic signaling-related processes following GR activation. Interestingly, AS animals also showed coordinated downregulation of ribosomal, proteasomal, and mitochondrial pathways, suggesting simultaneous suppression of protein synthesis and energy metabolism alongside activation of synaptic signaling programs. This pattern may represent a coordinated shift in transcriptional programs associated with synaptic signaling and cellular metabolism rather than a uniform activation of biosynthetic pathways, echoing findings that serotonergic activation in DRN enhances active coping [11,43].

Overall, these results indicate that GR activation in the DRN primarily modulates neuronal metabolic state and synaptic signaling, in contrast to the immune-dominated transcriptional response observed in the PFC.

### 3.4. Serotonergic System Under GR Activation

Despite the central role of the DRN as the source of forebrain serotonin, DEX-induced transcriptional changes in this region were limited to a small subset of serotonergic genes. Specifically, *Htr1d* expression increased in all groups, *Htr5b* decreased selectively in AS mice, and *Maoa* expression declined only in PS animals. These results suggest that GR activation selectively modulates serotonergic gene expression, primarily at the receptor and synaptic signaling level, rather than through large-scale changes in serotonin synthesis. This is consistent with previous reports showing that GR effects on DRN serotonergic neurons occur mainly via *Htr1a/Htr2a* receptor expression and synaptic plasticity rather than global modulation of serotonin production [4,44].

In the PFC, DEX-induced changes were more pronounced. *Htr1a* expression increased in both PS and AS mice, whereas *Htr2a* was upregulated in AS and control mice but remained unchanged in PS animals. *Bdnf* expression declined universally across all groups. These findings are in line with the idea that GR primarily influences postsynaptic receptor expression and synaptic plasticity in the PFC, with AS mice showing more coordinated receptor-gene upregulation, suggesting preserved plasticity and potential for active coping [4,28,30].

Functionally, stimulation of DRN serotonergic neurons promotes active coping, whereas inhibition increases anxiety-like behaviors [11]. Our transcriptional data suggest that enhanced receptor expression in AS mice (*Htr1a*, *Htr2a* in PFC, *Htr5b* in DRN) may reflect a predisposition for adaptive stress responses, whereas PS mice, with limited receptor changes and reduced Maoa, suggest reduced engagement of these regulatory programs after GR activation.

Pharmacological and stress studies highlight that serotonergic gene expression is highly context-dependent. For example, chronic lithium treatment in socially defeated mice upregulated *Tph2*, *Slc6a4*, *Htr1a*, and *Htr5b* in midbrain raphe nuclei, partially reversing stress-induced dysregulation [45]. Similarly, in MRNs of mice subjected to chronic social conflict, defeated animals exhibited *Tph2* downregulation and decreased *Htr3a*, while *Htr5b* was reduced in both defeated and winning mice [46]. These differences illustrate that stress, pharmacological interventions, and behavioral phenotypes can selectively engage receptor versus metabolic components of serotonergic signaling.

Importantly, our study focused on post-DEX transcriptional responses, without measuring serotonin release or DRN activity. Therefore, while transcriptional changes suggest phenotype-specific readiness of serotonergic networks, functional implications remain speculative, and discrepancies with other studies (different DEGs, stress models, or basal vs. stimulated conditions) emphasize the context-dependent nature of GR modulation.

Although our study focused exclusively on male mice, substantial evidence indicates that stress-related disorders exhibit pronounced sex differences at the behavioral, endocrine, and molecular levels. In both humans and animal models, females show a higher prevalence of stress-related disorders and differ from males in behavioral responses to stress, including anxiety- and depression-like behaviors, coping strategies, and fear-related responses, emphasizing that stress phenotypes cannot be directly extrapolated between sexes [47,48,49]. At the neuroendocrine level, female rodents generally display higher basal and stress-induced glucocorticoid secretion, slower recovery of HPA-axis activity following acute stress, and sex hormone-dependent modulation of glucocorticoid negative feedback, with estradiol playing a major role in shaping HPA-axis responsiveness [49,50]. Importantly, these functional differences are accompanied by molecular differences within HPA-axis regulatory circuits, including lower glucocorticoid receptor (GR) expression in the pituitary, hippocampus, and paraventricular nucleus, as well as sex-specific patterns of corticotropin-releasing hormone receptor (CRHR1/CRHR2) expression and signaling in stress-related brain regions [49]. Furthermore, experimental manipulation of corticosteroid receptors has demonstrated that GR- and mineralocorticoid receptor-dependent regulation of stress-related behaviors differs between males and females in a brain region-specific manner, suggesting that the neural mechanisms underlying HPA-axis feedback are themselves sexually dimorphic [50]. Therefore, the stress-response heterogeneity identified in the present study should be considered specific to male mice, and future studies will be required to determine whether comparable stress-response subgroups and their associated molecular signatures are also present in females.

This study has several limitations. The CSDS model represents a well-established paradigm for studying social stress-related neurobiological changes; however, it does not fully recapitulate the complexity of human affective disorders. In addition, only male mice were used, limiting the generalizability of the findings across sexes.

The pharmacological nature of the DEX challenge should be considered when interpreting the present findings. Unlike low-dose (0.1–0.2 mg/kg) dexamethasone suppression tests designed primarily to evaluate HPA-axis feedback sensitivity, the dose used here was selected to induce robust GR activation and characterize glucocorticoid-responsive transcriptional programs. The 6 h post-injection interval allowed assessment of both direct and downstream GR-mediated transcriptional responses, providing insight into differences in glucocorticoid sensitivity and regulatory capacity between control and chronically stressed animals.

An additional consideration is the circadian context of the dexamethasone challenge. HPA-axis activity and glucocorticoid responsiveness exhibit circadian variation, and previous studies using low-dose dexamethasone suppression tests have demonstrated time-dependent differences in corticosterone suppression [51]. Moreover, glucocorticoid-responsive transcription can be modulated by circadian mechanisms [51]. In the present study, a high pharmacological dose of DEX (2 mg/kg) was used to induce robust GR activation; therefore, although circadian influences on glucocorticoid signaling cannot be completely excluded, their contribution is expected to be limited under these conditions. Furthermore, DEX administration does not model a physiological stress response but rather serves as a pharmacological challenge to assess glucocorticoid sensitivity of gene expression programs. Therefore, the observed transcriptional effects should be interpreted in the context of HPA-axis reactivity rather than as direct stress-induced changes. Finally, pathway-level interpretations are based primarily on transcriptomic data and require further validation using complementary approaches, including targeted gene/protein expression analyses and functional assays.

## 4. Methods

### 4.1. Animals

Adult male mice of the C57BL/6 and CD1 strains were provided by the Center for Genetic Resources of Laboratory Animals at the Institute of Cytology and Genetics SB RAS, Novosibirsk, Russia (RFMEFI61914X0005). The animals were housed under standard conditions (12:12 h light/dark cycle, lights on at 8.00 a.m.; feed-pellets and water were available ad libitum). The mice were weaned at one month of age. Prior to CSDS, mice were group-housed (8–10 animals per cage) in cages measuring 36 × 23 × 12 cm according to institutional guidelines and GOST 33216-2014 [52]. Experiments were performed on mice 10–12 weeks of age [53]. All procedures were approved by the Ethics Committee of the Institute of Cytology and Genetics SB RAS (protocol 20.1, 11 March 2023). Animals were monitored daily throughout the experiment. No surgical procedures were performed and no analgesics were required. Bedding was changed regularly, and all efforts were made to minimize animal suffering and distress.

### 4.2. Experimental Design

C57BL/6 mice were used as experimental animals subjected to CSDS, whereas aggressive CD1 mice were used as resident aggressors during defeat sessions. The use of two different strains is based on the established CSDS paradigm, in which aggressive CD1 mice reliably induce social stress in susceptible C57BL/6 mice [7,18,54,55].

Male mice were randomly assigned to either the control or CSDS group. The CSDS group was exposed to agonistic confrontations for 30 consecutive days. Behavioral responses of defeated mice were assessed on day 29 of CSDS in the experimental cage previously used for agonistic interactions [53]. Behavioral parameters obtained during non-aggressive social contact were subsequently used for hierarchical clustering and subdivision of stressed animals into active coping strategy (AS) and passive coping strategy (PS) phenotypes (see Section 2.1). Intact control animals were housed individually for 5 days prior to the experiment.

A total of 24 h after the final stress session, mice received an intraperitoneal injection of either dexamethasone (2 μg/g body weight) or physiological saline at 9:00 a.m. This pharmacological dose was selected to induce robust and reproducible GR activation and facilitate detection of GR-dependent transcriptional responses [56]. Brain tissues were collected 6 h after DEX administration, a timepoint widely used in mouse dexamethasone suppression paradigms and suitable for assessing integrated glucocorticoid feedback, because it provides sufficient time for the development of both primary and secondary GR-dependent transcriptional responses and capturing downstream glucocorticoid-responsive molecular changes [5,35,36,37]. A total of 6 h after injection, animals were euthanized by rapid decapitation in accordance with the approved protocol and institutional guidelines and brain tissues were collected (Supplementary Methods S1).

To classify mice as passive (PS) or active (AS) coping-strategy phenotypes, we first performed hierarchical clustering on the complete behavioral dataset of all CSDS-exposed animals (n = 25). This initial exploratory analysis identified two animals with highly divergent behavioral profiles that formed isolated single-animal clusters, separate from the main population. Because these profiles were considered atypical and unrepresentative of the core PS/AS phenotypes, these two animals were excluded from further classification. The clustering procedure was then repeated on the remaining 23 animals, yielding 14 PS and 9 AS mice. Notably, the assignment of these 23 animals to the PS/AS clusters was unchanged from their grouping in the full dataset, confirming that the exclusion did not alter the clustering solution.

From each of the final PS and AS subgroups, 8 animals were randomly selected for downstream RNA-sequencing (total n = 16) and subsequently randomized to receive either saline or DEX (2 mg/kg) for further molecular analyses. All behavioral and transcriptomic data presented in this study are derived from these final 16 animals.

RNA was isolated from the prefrontal cortex and dorsal raphe nucleus, followed by RNA-seq library preparation for transcriptomic analysis. Sample sizes were determined based on previous studies using similar CSDS and transcriptomic designs [7,23,24,30]. No statistical power calculation was performed a priori. For RNA sequencing, 24 animals were included: 8 control, 8 PS, and 8 AS mice. Within each experimental group, animals were randomly assigned to receive either saline or DEX (2 mg/kg), resulting in four biological replicates per treatment condition. Following sequencing and initial quality assessment, DRN samples with *Tph2* expression values below the lower outlier threshold (Q1—1.5 × IQR) were excluded from downstream transcriptomic analyses, (1 from DEX-treated PS animals and 2 from DEX-treated AS animals). These samples were considered to have insufficient DRN tissue enrichment and were removed to minimize potential confounding effects associated with tissue collection quality. Final sample sizes for behavioral and transcriptomic analysis are indicated in Figure 1.

### 4.3. Chronic Social Defeat Stress

Prolonged experience of social defeat in male mice was induced using the sensory contact model [18] with some modifications [7]. Mice were housed in a steel cage (14 × 28 × 10 cm) bisected by a perforated transparent partition allowing the animals to see, hear, and smell each other but preventing physical contact. A C57BL/6 mouse was placed into the empty compartment adjacent to a CD1 mouse. The animals were left undisturbed for two days to adapt to new housing conditions and sensory contact before they were subjected to encounters. Every afternoon (14:00–17:00 p.m. local time) the partition was removed for 5 min to encourage aggressive interactions. To minimize the risk of injury and excessive stress, agonistic interactions were closely monitored and terminated immediately by reinstating the partition if intense aggression persisted for more than 1 min or if signs of severe distress or injury were observed. Animals were inspected daily throughout the experiment. During each defeat session, the C57BL/6 mouse was attacked by the aggressive CD1 mouse and showed defensive behavior (sideways postures, upright postures, withdrawal, or freezing). Once a day, after the defeat session, each C57BL/6 mouse was placed in an unfamiliar cage with a new aggressive CD1 mouse behind the partition, allowing continuous visual, olfactory, and auditory contact with the resident aggressor [7,18]. Each CD1 mouse remained in its original cage. Confrontation procedures were performed once a day for 30 days.

### 4.4. Behavior of the Defeated Mice During Social Defeat (Agonistic Confrontation Test)

Behavior in agonistic confrontation was assessed in an experimental cage where animals had previously experienced agonistic interactions. The partition separating the mice was removed, and the behavior of stressed males was recorded for 5 min. Scored behaviors included active defense, escape, freezing, social interest, passive sitting in the corner, bedding scattering, rearing, and grooming. Behavior was analyzed in three contexts relative to the aggressive partner: during attacks, during non-aggressive close social contact/proximity (≤2 cm), and at a distance (>2 cm). Proportions were calculated as percentages of total time in each context. Due to the non-normal distribution of behavioral variables, between-group comparisons were performed using two-sided Mann–Whitney U tests, with *p* < 0.05 considered statistically significant.

Cluster analysis of behavioral data was performed to separate defeated mice according to coping strategy into passive (PS) and active (AS) phenotypes. As victim behavior during attacks strongly depends on aggressor activity, clustering was based on the behavior of stressed animals during non-aggressive contact. Animals displaying extreme or atypical behavioral profiles were excluded from further analysis. Hierarchical clustering was performed in R using the hclust function (method = “average”) on scaled behavioral variables calculated with the dist function.

### 4.5. Brain Collection and RNA Isolation

The PFC was dissected immediately after brain extraction, snap-frozen in liquid nitrogen, and stored at −80 °C until further processing. DRN samples were isolated from cryosections using biopsy punches under cryostat conditions. Detailed tissue collection and dissection procedures are provided in Supplementary Methods S1.

Total RNA was isolated from frozen tissue samples using PureZOL reagent (Bio-Rad Laboratories, Hercules, CA, USA) for PFC samples and ExtractRNA reagent (Evrogen, Moscow, Russia) for DRN samples according to the manufacturers’ protocols. Purified total RNA was additionally cleaned using Agencourt RNAClean XP magnetic beads (Beckman Coulter, Brea, CA, USA).

RNA quantity and purity were assessed using a NanoDrop 2000 spectrophotometer (Thermo Fisher Scientific, Waltham, MA, USA) for PFC samples and a Qubit 2.0 fluorometer (Thermo Fisher Scientific, Waltham, MA, USA) with the Qubit RNA HS Assay Kit (Thermo Fisher Scientific, Waltham, MA, USA) for DRN samples. RNA integrity was evaluated using the Agilent 2100 Bioanalyzer (Agilent Technologies, Santa Clara, CA, USA) and the Total RNA Nano Kit (Agilent Technologies, Santa Clara, CA, USA). Only samples with an RNA integrity number (RIN) greater than 8.0 were used for transcriptomic analysis.

### 4.6. Library Preparation

Libraries for PFC and DRN were constructed and sequenced separately using the following protocol. In total, 24 samples for PFC and 21 samples for DRN were selected for genome-wide analysis of mRNA expression (RNA-seq). Strand-specific cDNA libraries of the PFC were prepared according to standard New England Biolab protocols (NEBNext Ultra II Directional RNA Library Prep, New England Biolabs, Ipswich, MA, USA). mRNA selection (polyA enrichment) was performed using magnetic beads with oligo-deoxy(T)25 (New England Biolabs, Ipswich, MA, USA). The DNA fragments size selection was carried out using AgencourtAMPure XP Beads (Beckman Coulter, Brea, CA, USA). Then, the resulting libraries were enriched by PCR (9 PCR cycles for PFC and 15 for DRN). The size and amount of the library was assessed by AgilentBioanalyzer, and then subjected to paired-end sequencing using the Illumina XTen platform for PFC and DNBSEQ-T7 for DRN samples (MGI Tech Co., Ltd., Shenzhen, China). RNA-seq library quality metrics are available in Supplementary Table S1. Since RNA sequencing libraries from the PFC and DRN were prepared and sequenced using different platforms and protocols, these datasets were analyzed separately and were not combined into a single differential expression dataset. This approach was used to minimize potential batch-related bias and to ensure comparability within each brain region.

### 4.7. Gene Expression Analysis

On average, 53 million (39–109 million) paired-end reads for PFC and 85 million (69–125 million) for DRN were obtained for each sample (Supplementary Table S1). Strand specificity was higher than 96% for each sample. The percentage of ribosomal bases was not higher than 2%.

Paired-end sequencing data were preprocessed by FASTP v0.20.1 [57], mapped to the reference mouse genome assembly mm10 (GRCm38) with HISAT2 v2.2.1 [58] software and quantified with featureCounts function from Subread package v2.0 [59] using gene reference annotation vM22.

Genes with ≥10 aligned reads in each sample were subjected to differential gene expression analysis in the DESeq2 R package v1.36.0 [60]. Differential gene expression analysis was performed separately for the PFC and DRN using a factorial design including treatment, stress phenotype, and their interaction. Stress effects were evaluated by comparing saline-treated PS and AS groups with saline-treated controls. DEX effects were assessed within each group by comparing DEX- and saline-treated animals. Interaction analysis was performed to identify genes whose response to DEX was modulated by stress phenotype, corresponding to the stress × dexamethasone interaction term in the DESeq2 factorial model. These genes are referred to as stress × DEX interaction DEGs. *p*-values were corrected for multiple comparisons using the Benjamini–Hochberg method, and genes with an adjusted *p*-value (padj) < 0.1 were considered differentially expressed.

Functional enrichment analysis was performed in R using the clusterProfiler (v.4.12.6) package. Gene Ontology (GO) over-representation analysis was conducted on subgroup-specific DEG using the enrichGO function with *Mus musculus* annotations from org.Mm.eg.db. To detect coordinated pathway-level changes, Gene Set Enrichment Analysis (GSEA) was performed on pre-ranked gene lists using the gseGO function. KEGG pathway enrichment analysis was performed using enrichKEGG function. To reduce redundancy among significantly enriched terms, GO term similarity reduction was performed using the simplify function in clusterProfiler, applying semantic similarity-based filtering of overlapping terms. Pathways with adjusted *p*-values (FDR) below 0.05 were considered significantly enriched. Complete enrichment analysis results are provided in Supplementary Tables S2–S4.

### 4.8. Cell-Type-Specific Enrichment

To assess cell-type-specific enrichment of DEGs, we used curated lists of cell-type-specific marker genes from [15], which were derived from published bulk tissue microarray data and single-cell RNA-sequencing data from brain regions. Cortical cell-type marker genes were used to analyze cell-type-specific expression in the PFC, while midbrain marker genes were used for the DRN. Enrichment of DEGs with cell-type-specific markers was assessed using odds ratio (OR) and Fisher’s exact test, with the background defined as all genes tested for differential expression. Analyses were performed with R version 4.4.1. OR and exact *p*-values for enrichment were obtained using the fisher.test() function from the stats package (included in base R).

## 5. Conclusions

Chronic social stress establishes phenotype- and region-specific baseline transcriptional states that shape downstream GR-dependent responses. In the PFC, AS mice exhibit limited baseline alterations but strong GR-induced transcriptional plasticity, particularly in translational and immune-related pathways, consistent with resilience-like adaptive capacity. PS mice display extensive baseline DEGs and pathway remodeling, yet show blunted GR responsiveness, reflecting stress-induced transcriptional saturation and vulnerability. In the DRN, GR activation produces more selective transcriptional changes, primarily involving synaptic- and metabolism-related pathways, with AS animals showing enhanced synaptic signaling and PS animals exhibiting suppression of neuronal projection genes. Collectively, these findings indicate that chronic stress configures distinct transcriptional landscapes that govern the magnitude, organization, and functional specificity of glucocorticoid responses across cortical and subcortical circuits.

## Data Availability

Raw and processed RNA-seq data generated in this study have been deposited in the Gene Expression Omnibus (GEO) under accession numbers GSE330674 (BioProject PRJNA846517) and GSE330675 (BioProject PRJNA1433139).

## Supplementary Materials

The following supporting information can be downloaded at: https://www.mdpi.com/article/10.3390/ijms27146442/s1. Reference [61] is cited in the Supplementary Materials.
