# Supplementary material for: Phenotype-Specific Transcriptomic Responses to Glucocorticoid Signaling in the Prefrontal Cortex and Dorsal Raphe Nucleus Following Chronic Social Stress
Source: Int J Mol Sci. 2026 Jul 20;27(14):6442. doi: 10.3390/ijms27146442 (PMC13410218; doi:10.3390/ijms27146442)
Supplement: Supplementary file 1 [file ijms-27-06442-s001.zip › Supplementary_Methods_S1.pdf]

Tissue collection

The prefrontal cortex (PFC) was dissected immediately after brain extraction and stored at −80 °C until further use. For PFC isolation, the olfactory bulbs were first removed, after which the cortical region spanning approximately 0.75 to 3.25 mm anterior to bregma was dissected at an angle of approximately 30°. The resulting teardrop-shaped tissue block included the secondary motor cortex, anterior cingulate cortex, orbital cortex, prelimbic cortex, and infralimbic cortex, all considered components of the murine prefrontal cortex ([61] Carlén, 2017) (Figure S1).

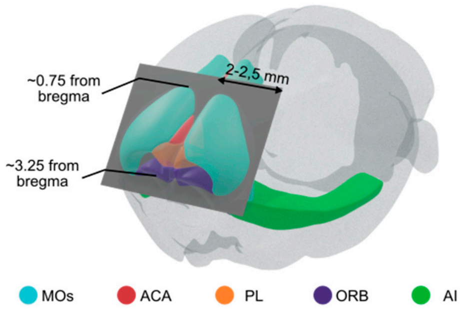

MOs - secondary motor cortex
ACC - anterior cingulate cortex
PL - prelimbic cortex
IL - infralimbic cortex
ORB - orbital cortex
AI - agranular insular cortex

***Figure S1**. Murine PFC structure* Brain samples containing the dorsal raphe nuclei (DRN) were embedded in OCT compound (optimal cutting temperature compound), frozen in liquid nitrogen, and stored at −70 °C until processing. The DRN region was isolated according to the coordinates of the Allen Mouse Brain Atlas, corresponding to 4.240-4.655 mm posterior to bregma (coronal levels 96-100). Depending on the rostrocaudal level, the target area ranged from approximately 0.5 × 0.5 mm to 1 × 1 mm (Figure S2). Control cryosections were prepared to verify the anatomical localization of the structures. Subsequently, 3-4 cryosections (100 μm thick) were obtained from each adult mouse brain using a cryostat. Tissue samples were collected using a Sample Corer biopsy punch (Fine Science Tools) with an inner diameter of 0.5 mm. All procedures were performed at −20 °C within the cryostat chamber. Collected tissue samples were immediately transferred into ExtractRNA reagent (Evrogen, Russia), and RNA isolation was performed immediately thereafter.

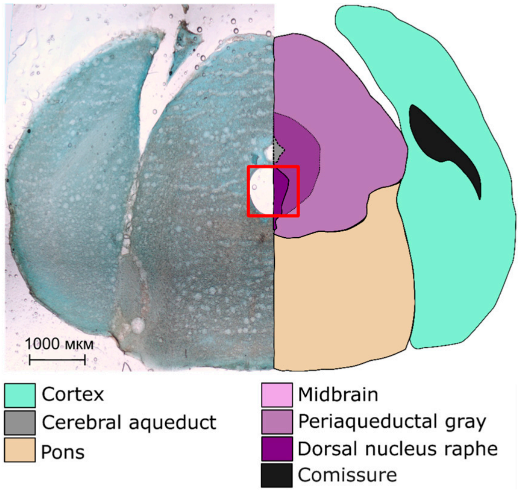

Figure S2. *Structure of the mouse dorsal raphe nuclei (DRN) and the region used for tissue collection. The left panel shows a representative microscopic image, whereas the right panel presents a schematic illustration. The red box indicates the DRN area dissected for the study*
